# Decreased K5 receptor expression in the retina, a potential pathogenic mechanism for diabetic retinopathy

**Published:** 2012-02-04

**Authors:** Jianfang Ma, Chaoyang Li, Chunkui Shao, Guoquan Gao, Xia Yang

**Affiliations:** 1 Department of Medicine, PLA 476th Hospital, Fuzhou, China; 2 Department of Biochemistry, Zhongshan School of Medicine, Sun Yat-sen University, Guangzhou, Guangdong Province, China; 3 State Key Laboratory of Ophthalmology, Zhongshan Ophthalmic Center, Sun Yat-sen University, Guangzhou, China; 4 Department of Pathology, The Third Affiliated Hospital, Sun Yet-sen University, Guangzhou, China; 5 China Key Laboratory of Tropical Disease Control, Sun Yat-sen University, Ministry of Education, Guangzhou, China; 6 Key Laboratory of Functional Molecules from Marine Microorganisms, Sun Yat-sen University, Department of Education of Guangdong Province, Guangzhou, China

## Abstract

**Purpose:**

Plasminogen kringle 5 (K5) is a potent angiogenic inhibitor and specifically binds to the voltage-dependent anion channel believed to function as the K5 receptor (K5R). To investigate the role of K5R in diabetic retinopathy, the present study measured the expression levels of K5R in the retina of diabetic retinopathy models. In cultured retinal Müller cells, K5 inhibited vascular endothelial growth factor (*VEGF*) expression as shown with enzyme-linked immunosorbent assay and western blot analysis, suggesting that K5 has a direct effect on Müller cells.

**Methods:**

To identify K5R in retinal Müller cells, ligand binding and competition assays as well as real-time reverse transcriptional polymerase chain reaction were performed in Müller cells. ^125^I-K5 showed saturable binding to cultured Müller cells. The binding can be competed off by an excess amount of unlabeled K5 but not by angiostatin, demonstrating the specificity of the K5 binding to Müller cells. Consistent with the binding assay, reverse transcriptional polymerase chain reaction using voltage-dependent anion channel–specific primers detected the K5R mRNA in the Müller cells.

**Results:**

Interestingly, *K5R* mRNA expression in Müller cells was downregulated by diabetic conditions including hypoxia and high glucose medium. Incubation with K5 ligand prevented hypoxia-induced downregulation of *K5R*. Furthermore, *K5R* expression was also downregulated in the retina of the oxygen-induced retinopathy model, a model of ischemia-induced retinal neovascularization. In a type 1 diabetic rat model, *K5R* expression in the retina was significantly suppressed in rats that had diabetes for 5 and 8 weeks.

**Conclusions:**

These results suggest that *K5R* is expressed in retinal Müller cells, which may mediate the inhibitory effect of K5 on *VEGF* expression. In diabetes conditions, K5R expression levels are decreased in the retina, which could contribute to the *VEGF* overexpression in diabetic retinopathy. These findings suggest that the decreased levels of K5R may also play a pathogenic role in diabetic retinopathy.

## Introduction

Proliferative diabetic retinopathy (PDR) is characterized by retinal neovascularization (NV), a major cause of blindness [1]. Several endogenous angiogenic inhibitors as well as proangiogenic factors exist in ocular tissues such as the retina and vitreous [2,3]. Endogenous angiogenic inhibitors and proangiogenic factors are in a delicate balance that regulates angiogenesis. Under certain pathological conditions such as PDR, endogenous angiogenic inhibitors have been found to decrease while the proangiogenic factors increase in the retina and vitreous, leading to a disturbed balance in angiogenic control and consequently retinal NV [4–6].

Plasminogen kringle 5 (K5), a proteolytic fragment of plasminogen, is a potent angiogenic inhibitor [7]. Previous studies have shown that K5 inhibits endothelial cell (EC) proliferation and migration and induces EC apoptosis [7,8]. Intravitreal injection of K5 inhibited retinal NV and reduced retinal vascular leakage in diabetic retinopathy (DR) models [9]. Furthermore, K5 and its deletion fragment also displayed inhibitory effects on tumor growth via blocking angiogenesis [10,11]. Adeno-associated virus-mediated delivery of K5 was found to inhibit growth of ovarian cancer and tumor NV [12]. A recent study reported that nanoparticle-mediated *K5* gene delivery had sustained inhibitory effects on retinal vascular leakage in diabetic rats and ischemia-induced retinal NV [13]. Therefore, K5 is believed to be a promising therapeutic peptide for treating DR as well as solid tumor [13]. Our previous studies showed that K5 attenuates hypoxia-induced vascular endothelial growth factor (*VEGF*) overexpression in vascular cells [14]. However, the molecular mechanism for K5′s antiangiogenic effects has not been elucidated.

Researchers reported previously that voltage-dependent anion channels (VDACs) are expressed on the cell surface of EC [15]. Furthermore, K5 has been shown to bind to VDAC with high specificity and affinity in EC, correlating with K5′s antiproliferative activities in cultured EC. The binding of K5 to EC can be blocked by antibodies specific for VDAC. Based on these findings, VDAC has been proposed as the K5 receptor (K5R), mediating its antiangiogenic effects [15]. However, the role of K5R in angiogenic disorders and PDR has not been established. The cellular localization of K5R in the retina has not been defined.

Retinal Müller cells are the major producer of proangiogenic factors such as VEGF in DR and play a key role in retinal NV [3,16]. Since K5 inhibits *VEGF* expression under hypoxia, we hypothesize that K5 directly inhibits angiogenic factor production from retinal Müller cells in diabetic conditions. The present study identified K5R in retinal Müller cells and determined the expression levels of K5R under diabetic conditions.

## Methods

### Materials and cell line

Expression and purification of recombinant K5 were performed as described previously [14]. The K5/pET22 construct was introduced into *E. coli* strain BL-21/DE3 (Novagen, Madison, WI). This vector provides a signal peptide that enables the recombinant protein to enter the periplasmic space. The expression and purification followed the protocol recommended by Novagen with some modifications. Briefly, expression was induced by the addition of isopropylthio-β-galactoside (IPTG) and performed for 10 h at 25 °C. Periplasmic proteins were released by digestion with lysozyme and separated from cells by centrifugation. K5 was purified by passing through the His-Bind column (Novagen). The purity and identity of recombinant K5 was examined by SDS–PAGE and western blot analysis using an antibody specific to His-tag (Oncogene Research Products, Cambridge, MA).The rat Müller cell line, rMC-1, a kind gift from Dr. Vijay Sarthy at Northwestern University, was cultured in low glucose Dulbecco's Modified Eagle's Medium (DMEM; Gibco BRL, Gaithersburg, MD) containing 10% fetal bovine serum (FBS; Invitrogen, Carlsbad, CA) [17]. The cultured cells were exposed to media containing 1% FBS for 4 h before proteins were added.

### Enzyme-linked immunosorbent assay and western blot analysis of vascular endothelial growth factor

Müller cells were seeded in 100-mm dishes and cultured in a CO_2_ incubator to reach 60%–70% confluence. The cells were washed three times with PBS (pH 7.4, 8g NaCl, 0.2g KCl, 3.628 g Na_2_HPO_4_•12H_2_O, 0.24 g KH_2_PO_4_ metered volume to 1,000 ml with distilled water), and the growth medium replaced with a serum-free DMEM and exposed to 1% oxygen. K5 was added to the medium in various concentrations (50, 100, 200, 400, and 800 nM) and incubated with the cells for 24 h. The cells cultured under normoxia were used for control. The cells were harvested, and the protein concentration was measured with the BioRad protein assay (Bio-Rad, Hercules, CA). Equal amounts of total cellular protein (35 μg) from each group were used for western blot analysis using an anti-VEGF antibody with the ECL Detection System (Amersham International plc, Piscataway, NJ). The same membrane was stripped and reblotted with an antibody specific to β-actin. VEGF secreted into the culture medium was measured with an enzyme-linked immunosorbent assay (ELISA) kit (R&D Systems, Inc., Minneapolis, MN) specific for VEGF.

### Binding of ^125^I-labeled K5 to Müller cells

K5 was labeled with ^125^I using the Chloromine T ^125^I Labeling Kit (ICN Pharmaceuticals, Inc. Costa Mesa, CA) following a protocol recommended by the manufacturer. For the binding assay, Müller cells were seeded in 12-well plates and cultured until confluence. Cells were washed, and the culture medium was replaced with binding buffer (PBS containing 3 mM CaCl_2_, 1 mM MgCl_2_, and 5 mg/ml BSA). ^125^I-K5 was added to various concentrations (0, 6.25, 12.5, 25, 50, 75, 100, 150, 200, and 250 nM) and incubated with the cells for 1 h with gentle shaking at 4 °C. The medium was removed, and cells washed three times with PBS. The cells were then lysed by adding 0.35 ml 10% sodium dodecyl sulfate. The cell lysates were collected, and the ^125^I-K5 bound to Müller cells was quantified with a gamma counter (Perkin Elmer, Watham, MA).

### Competition with ^125^I-K5 for binding to Müller cells by unlabeled K5 or angiostatin

The cells were incubated with 50 nM ^125^I-K5 in the presence of increasing concentrations (0, 50, 250, 1,250, and 6,250 nM) of unlabeled K5 or angiostatin as described above. After washing, the bound ^125^I-K5 was quantified.

### Streptozotocin-induced diabetes

Brown Norway (BN) rats were purchased from Harlan (Indianapolis, IN). Care, use, and treatment of all animals in this study were in strict agreement with the Association for Research in Vision and Ophthalmology Statement for the Use of Animals in Ophthalmic and Vision Research. Eight-week-old BN rats were given a single intravenous injection of STZ (50 mg/kg in 10 mmol/l of citrate buffer, pH 4.5) after an overnight fasting. Control rats received injections of citrate buffer alone. Serum glucose levels were checked 24 h after STZ injection and every 2 days thereafter, and only the animals with glucose levels higher than 350 mg/dl were considered diabetic [18,19].

### Oxygen-induced retinopathy

Induction of retinal neovascularization was performed as described by Smith et al. [20], with minor modifications. Briefly, newborn BN rats at P7 were exposed to hyperoxia (75% O_2_) for 5 days (P7–12) and then returned to normoxia (room air) to induce retinal neovascularization. Control rats were maintained in constant room air.

### Real-time reverse transcription–polymerase chain reaction

Real-time reverse transcription–polymerase chain reaction (RT–PCR) was performed as described previously [21]. The primers used for human *VDAC1* (5′-AAC ACT CGC TTT GGA ATA AC-3′ and 5′-AGT CCT AAA CCA AGC TTG TG-3′) amplified a 180-bp single-band product. The 18S rRNA was amplified using primers (5′-TGC TGC AGT TAA AAA GCT CGT-3′, and 5′-GGC CTG CTT TGA ACA CTC TAA-3′) to normalize the *K5R* mRNA levels.

### Statistical analysis

The Student *t* test was used in all statistical analyses. A p value of less than 0.05 was considered statistically significant.

## Results

### K5 attenuated the hypoxia-induced vascular endothelial growth factor overexpression in cultured Müller cells

**Figure 1:**
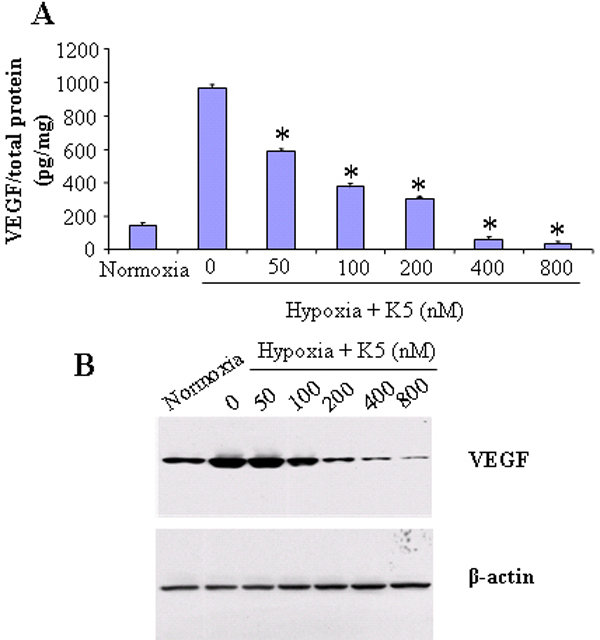
K5 inhibited the hypoxia-induced vascular endothelial growth factor overexpression in cultured Müller cells. rMC-1 cells were exposed to normoxia (20% oxygen) and hypoxia (1% oxygen) with different concentrations of K5 for 24 h. **A**: VEGF secreted to the conditioned medium was measured using ELISA specific for VEGF, normalized by total protein concentration and expressed as a picogram of VEGF per milligram of total protein (mean±SD, n=4). Values significantly lower than control are indicated (*p<0.05). **B**: VEGF levels in cell lysates were measured with western blot analysis using 50 µg cellular proteins. The membrane was stripped and then reblotted with an anti-β-actin antibody.

Overexpression of *VEGF* in the retina induced by hypoxia plays a key role in retinal NV [6]. Since retinal Müller cells are the major source of VEGF in DR, we determined whether K5 has a direct inhibitory effect on *VEGF* expression in Müller cells. The rat Müller cell line, rMC-1, was exposed to 1% oxygen to induce hypoxia. As shown with enzyme-linked immunosorbent assay (ELISA) specific for VEGF, the secreted VEGF in the conditioned medium was significantly induced by hypoxia, and this induction was attenuated by K5 in a concentration-dependent manner (Figure 1A). Western blot analysis showed that K5 also blocked the increase in cellular VEGF levels induced by hypoxia in Müller cells (Figure 1B).

### Detection of K5R expression in retinal Müller cells

**Figure 2:**
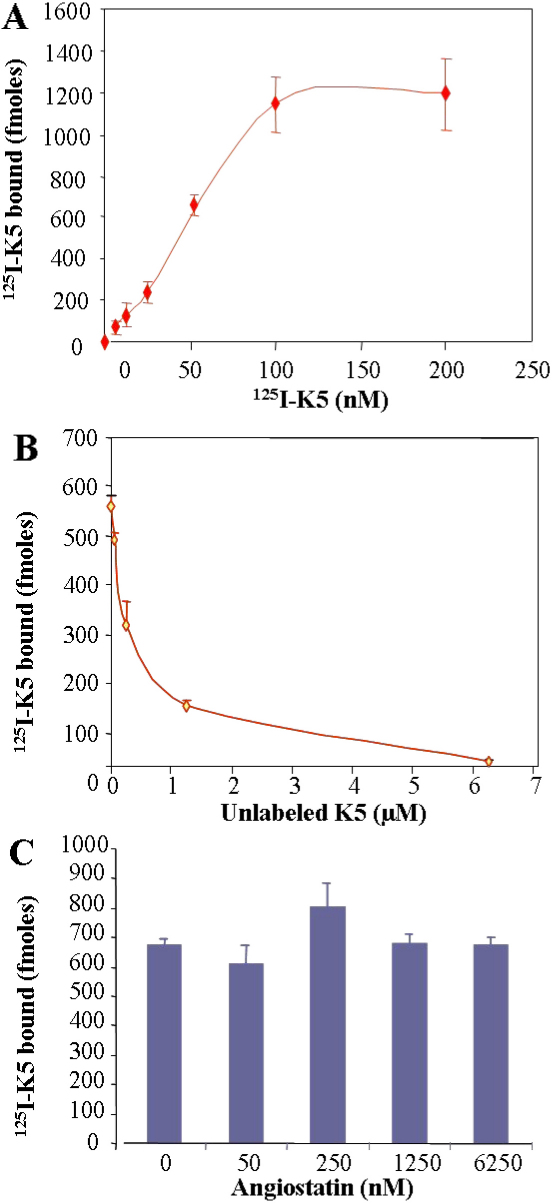
K5 specifically bound to retinal Müller cells. **A**: Rat Müller cells were incubated with increasing concentrations of ^125^I-labeled K5 for 1 h followed with thorough washing with PBS. Then ^125^I-K5 bound to the cells were quantified using a γ-counter (mean±SD, n=3). The binding of ^125^I-K5 to Müller cells appeared concentration-dependent and saturated above 100 nm of K5. **B**: The cells were incubated with 50 nM of ^125^I- K5 and increasing concentrations of unlabeled K5 at 37 °C for 1 h, washed with PBS, and ^125^I-K5 on the cells was quantified by γ-counting (mean±SD, n=3). **C**: The cells were incubated with 50 nM of ^125^I- K5 and increasing concentrations of unlabeled angiostatin under the same conditions. After thorough washing, ^125^I-K5 on the cells were quantified (mean±SD, n=3). The binding of ^125^I-K5 to Müller cells was competed off by increasing concentrations of unlabeled K5 but not by angiostatin.

To test the hypothesis that the effect of K5 in Müller cells is mediated by a receptor for K5, we used a K5 binding assay. The cultured rMC-1 cells were incubated with increasing concentrations of ^125^I-K5. After unbound ^125^I-K5 was removed by washing, the cells were lysed, and ^125^I-K5 bound to the cells were quantified using a gamma counter, which showed a concentration-dependent and saturable binding of ^125^I-K5 to Müller cells (Figure 2A). We calculated the K_d_ as 31 nM, comparable to that reported by Gonzalez-Gronow et al. [15].

To further demonstrate the specificity of the K5 binding, the Müller cells were incubated with 50 nM ^125^I-K5 in the presence of excess amounts of unlabeled K5 or unlabeled angiostatin, kringles 1–4 of plasminogen [22]. The results showed that binding of ^125^I-K5 on the Müller cells was competed off by excess amounts of unlabeled K5 but not by angiostatin (Figure 2B,C). The unlabeled K5 inhibited the binding of ^125^I-K5 in a concentration-dependent manner, demonstrating the specificity of the binding of ^125^I-K5 to Müller cells (Figure 2B). These results suggest that K5R is expressed in retinal Müller cells.

### K5R expression was downregulated by hypoxia and high glucose in Müller cells

**Figure 3:**
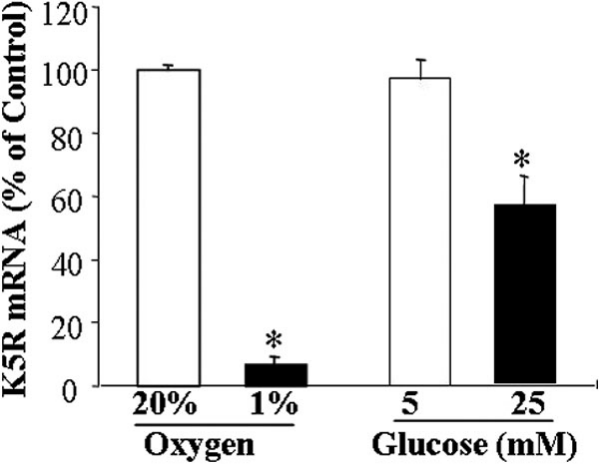
K5R expression was down-regulated by hypoxia and high glucose. Müller cells were exposed to hypoxia (1% oxygen) or normoxia (20% oxygen) at 37 °C for 12 h. The cells were cultured in high glucose (25 mM glucose) and low glucose (5 mM glucose + 20 mM mannitol) media for 24 h. Total RNA was isolated from the cells and used for real-time RT–PCR of *K5R*, normalized by the 18s RNA level and expressed as % of respective control (mean±SD, n=3). Values significantly lower than control are indicated (*p<0.05). Hypoxia and high glucose significantly downregulated the expression of the *K5R* mRNA in cultured Müller cells.

Previous studies have shown that the expression of *VEGF* is upregulated in retinal Müller cells [16]. Since K5 inhibits *VEGF* expression under hypoxia in Müller cells, we determined whether *K5R* expression is altered under diabetic conditions, which may contribute to the overexpression of *VEGF*. We compared expression levels of the *K5R* mRNA in the cells exposed to high glucose medium (25 mM glucose) with those in low glucose medium (5 mM glucose + 20 mM mannitol control). Real-time RT–PCR using specific primers for *K5R* showed that *K5R* mRNA levels were significantly decreased by high glucose exposure, compared to that in the low glucose medium (Figure 3).

Similarly, the cells were exposed to 1% oxygen to induce hypoxia, and the *K5R* mRNA was quantified and compared to that in the normoxia control. Real-time RT–PCR showed that *K5R* mRNA levels were also significantly decreased by hypoxia (Figure 3).

### K5 attenuated the downregulation of *K5R* expression

**Figure 4:**
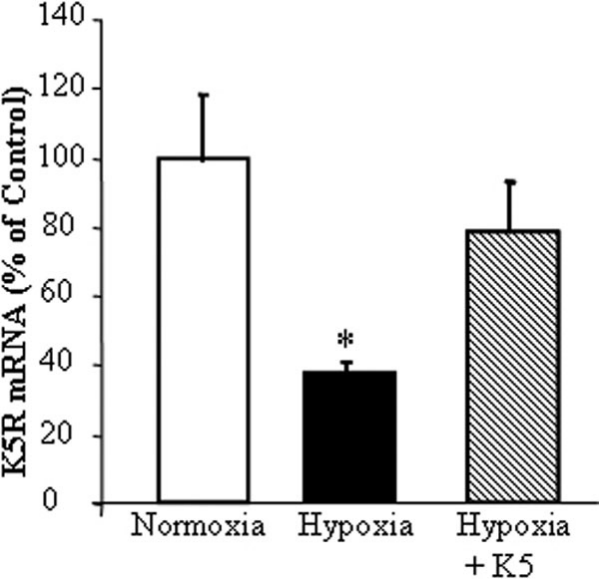
K5 induced K5R expression in Müller cells. Müller cells were exposed to 1% oxygen in the presence of 200 nM K5 at 37 °C for 12 h. Total RNA was isolated from the treated cells. The K5R mRNA was quantified with real-time RT–PCR and normalized by 18s RNA levels (mean±SD, n=3). The copies of K5R mRNA in hypoxia group were significantly lower than that in normoxia group (*p<0.05). K5 prevented the hypoxia-induced downregulation of K5R mRNA expression in cultured Müller cells.

To determine the impact of K5 on the expression of *K5R*, Müller cells were exposed to hypoxia in the presence of purified recombinant K5. As shown with real-time RT–PCR, hypoxia significantly decreased *K5R* mRNA levels, while adding the K5 peptide attenuated the hypoxia-induced downregulation of *K5R* (Figure 4). This observation suggests that preventing the decrease in *K5R* by K5 may represent a new mechanism of its action.

### *K5R* expression was downregulated in the retina of the oxygen-induced retinopathy rat model

**Figure 5:**
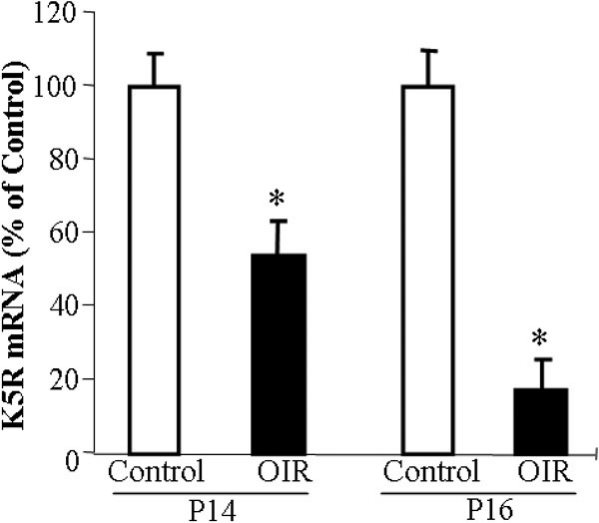
K5R expression was down-regulated in the retina of the oxygen-induced retinopathy rat model. Rats were exposed to 75% oxygen from P7 to P12 of age and then returned to room air at P12. Total RNA was isolated from the retina of the OIR rats at P14 and P16. mRNA levels of *K5R* were quantified with real-time RT–PCR and normalized with 18s RNA levels and expressed as % of that in age-matched normal rats (mean±SD, n=3). Values significantly lower than control are indicated (*p<0.05). *K5R* mRNA levels were significantly decreased in the retina of the OIR models, compared to that in normal rats.

To investigate whether K5R is also downregulated in the retina of DR models, we employed rats with oxygen-induced retinopathy (OIR), a commonly accepted model for PDR [20,23]. At age of P14 and P16, the OIR rats showed significantly decreased *K5R* mRNA levels in the retina, compared to those of age-matched rats at constant normoxia (Figure 5). This finding suggests that the *K5R* decline in this model may be another pathogenic mechanism for retinal NV in OIR rats.

### *K5R* expression is suppressed in the retina of diabetic rats

**Figure 6:**
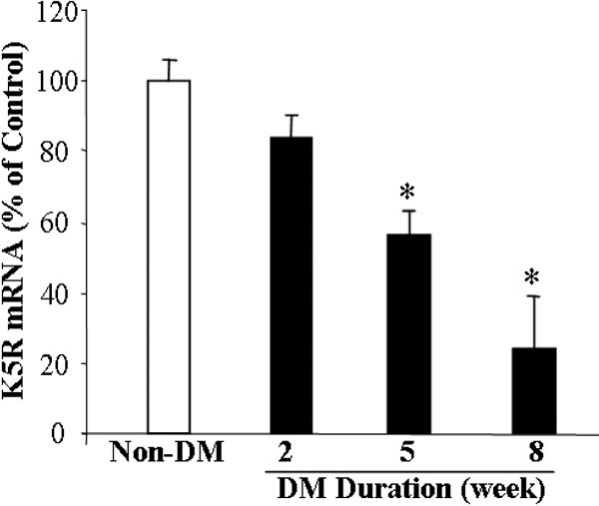
K5R expression is suppressed in the retina of diabetic rats. Diabetes was induced in adult rats with an injection of STZ and monitored by blood glucose levels. The rats with blood glucose levels higher than 350 mg/dl were used as diabetic rats. Retinas were isolated from diabetic rats at 2, 5, and 8 weeks after the STZ injection. Retinal mRNA levels of *K5R* were quantified with real-time RT–PCR and normalized by the 18s RNA level. The normalized *K5R* mRNA levels were expressed as % of that in non-diabetic control (mean±SD, n=3). Values significantly lower than control are indicated (*p<0.05). Diabetic rats showed significantly reduced *K5R* mRNA levels in the retina, compared to non-diabetic rats.

*K5R* expression was also measured in the retina of STZ-induced diabetic rats, a type 1 diabetes model. Real-time RT–PCR showed that *K5R* mRNA levels in the retina declined in rats that had diabetes for 5 and 8 weeks, compared to that in the non-diabetic control. The decrease of the *K5R* mRNA levels appeared to be dependent on the duration of diabetes (Figure 6), suggesting that the decrease in *K5R* may also contribute to retinal vascular leakage in this model.

## Discussion

Previous studies have shown that endogenous angiogenic inhibitors are downregulated in the vitreous from patients with DR and in the retina of DR models [4,5]. The decrease of angiogenic inhibitors and overexpression of proangiogenic factors disturb the balance of angiogenesis regulation, leading to DR [23,24]. The present study showed for the first time that the receptor for the angiogenic inhibitor K5 is also downregulated in the retina of DR models, suggesting that decreased expression of receptors for angiogenic inhibitors may weaken the antiangiogenic action and, thus, represents a new pathogenic mechanism for DR.

Several groups have independently demonstrated that K5 is a potent angiogenic inhibitor, as it inhibits EC proliferation and migration [7,9]. Toward its mechanism of action, Gonzalez-Gronow et al. [15] identified VDAC1 as the receptor for K5, which is expressed on the surface of EC. The expression of K5R on EC can explain the direct inhibitory effect of K5 on EC proliferation and migration [15]. Our previous studies have shown that K5 inhibits *VEGF* overexpression in the retina of DR models [14]. Retinal Müller cells play a key role in DR, as they are the major source of inflammatory and angiogenic factors such as VEGF [16,25]. Our results showed that K5 directly inhibits *VEGF* expression in cultured Müller cells. The mechanism for the direct effect of K5 on *VEGF* expression in Müller cells is unclear. To investigate whether the receptor mediates the K5 effects on Müller cells, we performed a ^125^I-K5 binding assay. The assay demonstrated that K5 has saturable and specific binding to cultured Müller cells. The binding of K5 is reversible and can be competed off by excess amounts of unlabeled K5. RT–PCR using *K5R*-specific primers detected *K5R* mRNA in cultured Müller cells. These results suggest that *K5R* is expressed in Müller cells. Interestingly, the K5 binding to K5R is not competed off by angiostatin, kringles 1–4 of plasminogen, suggesting that K5R is specific for K5 and not the receptor for plasminogen or for kringles 1–4. Expression of *K5R* in Müller cells could explain the direct effect of K5 on Müller cells in downregulation of *VEGF* expression.

To study whether *K5R* expression is altered in retinal Müller cells under diabetic conditions, we exposed the cells to hypoxia and high glucose medium, as hypoxia and high glucose are the major causative factors for retinal inflammation and NV in DR, as both have been shown to induce expression of proangiogenic and proinflammatory factors [20]. Our results showed that hypoxia and high glucose concentration downregulate *K5R* expression. The weakened effect of K5 due to the decrease in its receptor in diabetic conditions may contribute to the overexpression of *VEGF* in Müller cells under diabetic conditions.

In this study, rMC-1 was used as an in vitro model to study the expression of *K5R*. rMC-1 is a widely used Müller cell line, since this line is derived from rat Müller cells and expresses Müller cell markers [26,27]. Using the cloned cell line excludes potential confusion from contamination of other cell types in primary Müller cell culture. However, the changes in *K5R* observed in this cell line remain to be confirmed in Müller cells in the retina in the future.

STZ-induced diabetes is a commonly used model for non-proliferative DR, as this model develops retinal inflammation and vascular leakage but not NV [18]. The OIR model develops preretinal NV induced by ischemia [20]. Although the OIR model is not a diabetic model, this model is commonly used as a model for PDR, as the pathological changes and pathogenic mechanism are similar to PDR [20]. Our in vivo results showed that K5R levels in the retina decreased in both models. Since K5 has anti-inflammatory, antipermeability, and antiangiogenic activities, the decreased K5R may contribute to retinal vascular leakage and NV in these models. The decreased K5R may represent a new mechanism leading to the weakened antiangiogenic actions of the endogenous angiogenic inhibitor and disturbing the balance between proangiogenic and antiangiogenic systems. Therefore, upregulation of *K5R* may become a new, promising therapeutic strategy for DR.
